# Supplementary material for: Effectiveness of specific stabilization exercise compared with traditional trunk exercise in women with non-specific low back pain: a pilot randomized controlled trial
Source: PeerJ. 2020 Nov 27;8:e10304. doi: 10.7717/peerj.10304 (PMC7703373; doi:10.7717/peerj.10304)
Supplement: Supplemental Information 2 [file peerj-08-10304-s002.pdf]

ClinicalTrials.gov Search Results 05/10/2020

	NCT Number	Title	Other Names	Status	Conditions	Interventions	Characteristics	Population	Sponsor/ Collaborators	Funder Type	Dates	Locations
1	NCT02103036	Valuation of the Low Back Pain Treated With Different Types of Active Exercises in Women<br>Study Documents:	Title Acronym: ECOLUM<br>Other Ids: ECOLUM	Unknown status	• Low Back Pain	• Procedure: Core Stability Exercises (CSE)<br>• Procedure: Traditional Back School<br>• Device: TENS<br>• Device: Infra-red	Study Type: Interventional<br>Phase: Not Applicable<br>Study Design:<br>• Allocation: Randomized<br>• Intervention Model: Parallel Assignment<br>• Masking: Single (Participant)<br>• Primary Purpose: Treatment<br>Outcome Measures:<br>• Changes in Visual Scale Analogue (VAS)<br>• Changes in the Roland-Morris Disability Questionnaire	Enrollment: 30<br>Age: 18 Years to 70 Years (Adult, Older Adult)<br>Sex: Female	• University Rovira i Virgili<br>• Hospital Universitari Sant Joan de Reus	• Other	Study Start: March 2014<br>Primary Completion: March 2015<br>Study Completion: October 2015<br>First Posted: April 3, 2014<br>Results First Posted: No Results Posted<br>Last Update Posted: December 5, 2014	• Faculty of Medicine and Health Sciences, Reus, Tarragona, Spain
